# Emerging Roles of Extracellular Vesicles in Pneumococcal Infections: Immunomodulators to Potential Novel Vaccine Candidates

**DOI:** 10.3389/fcimb.2022.836070

**Published:** 2022-02-14

**Authors:** Saba Parveen, Karthik Subramanian

**Affiliations:** Host-Pathogen Laboratory, Pathogen Biology Division, Rajiv Gandhi Centre for Biotechnology, Thiruvananthapuram, India

**Keywords:** *Streptococcus pneumoniae* (pneumococcus), extracellular vesicles (EVs), immunomodulation, bacterial pathogenesis, inflammation, acellular vaccines, diagnostic biomarkers

## Abstract

The Gram-positive bacterial pathogen, *Streptococcus pneumoniae* is a major global health threat that kills over one million people worldwide. The pneumococcus commonly colonizes the nasopharynx asymptomatically as a commensal, but is also capable of causing a wide range of life-threatening diseases such as pneumonia, meningitis and septicemia upon migration into the lower respiratory tract and spread to internal organs. Emergence of antibiotic resistant strains and non-vaccine serotypes has led to the classification of pneumococcal bacteria as a priority pathogen by the World Health Organization that needs urgent research into bacterial pathogenesis and development of novel vaccine strategies. Extracellular vesicles are spherical membrane bound structures that are released by both pathogen and host cells, and influence bacterial pathogenesis as well as the immune response. Recent studies have found that while bacterial vesicles shuttle virulence factors and toxins into host cells and regulate inflammatory responses, vesicles released from the infected host cells contain both bacterial and host proteins that are antigenic and immunomodulatory. Bacterial membrane vesicles have great potential to be developed as cell-free vaccine candidates in the future due to their immunogenicity and biostability. Host-derived vesicles isolated from patient biofluids such as blood and bronchoalveolar lavage could be used to identify potential diagnostic biomarkers as well as engineered to deliver desired payloads to specific target cells for immunotherapy. In this review, we summarize the recent developments on the role of bacterial and host vesicles in pneumococcal infections and future prospects in developing novel therapeutics and diagnostics for control of invasive pneumococcal diseases.

## Introduction

*Streptococcus pneumonia*e (the pneumococcus) is a leading cause of mortality and morbidity worldwide due to community-acquired pneumonia and its complications (GBD 2016 Lower Respiratory Infections Collaborators, 2018), out of which the majority are young children below 5 years (McAllister et al., 2019). Although healthy people can be asymptomatically colonized by pneumococci, bacterial invasion into the lower respiratory tract typically causes severe diseases such as pneumonia, septicemia, meningitis and otitis media particularly among the elderly, children and the immunocompromised. Pneumococcal strains resistant to beta-lactams and macrolides have emerged due to antibiotic misuse and horizontal transmission of antibiotic-resistance genes (Cherazard et al., 2017). The spread of pneumococcal resistant clones worldwide is a major global concern. Consequently, the pneumococcus has been declared by the World Health Organization as one of the top priority pathogens that urgently requires novel antimicrobial strategies. Although pneumococcal polysaccharide and conjugate vaccines have reduced the overall disease burden, they only offer limited protection against few capsular serotypes (Daniels et al., 2016). Serotype replacement by non-vaccine serotypes is a serious problem that needs immediate attention. Hence, it is important to develop vaccines that offer broad range protection against several serotypic variants.

Extracellular vesicles are nanometer to micrometer sized spherical particles released by organisms across several kingdoms spanning archaea, prokaryotes as well as eukaryotes (Deatherage and Cookson, 2012). During the course of an infection, both pathogen- and host-derived vesicles are produced and play important roles in determining the course of the infection. Bacterial vesicles are 20-250 nm in diameter that are released by both Gram-negative and Gram-positive bacteria and shuttle proteins, carbohydrates, lipids, nucleic acids, and other virulence factors to host cells (Kim et al., 2015). Consequently, bacterial vesicles are gaining increasing attention due to their potential role in regulating bacterial virulence properties and the host immune response (MacDonald and Kuehn, 2012, Kaparakis-Liaskos and Ferrero, 2015). Moreover, bacterial vesicles have great potential as acellular vaccines since they express immunogenic bacterial antigens and are safer compared to their live infectious counterparts (Acevedo et al., 2014).

Host-derived extracellular vesicles (EVs) vary in size between 30-5000 nm and are classified into three major subtypes, namely exosomes, microvesicles and apoptotic bodies, based on their size and biogenesis (Schorey et al., 2015). While exosomes are of endo-lysosomal origin, microvesicles are generated by outward budding from plasma membrane and apoptotic bodies are products of apoptotic cell disassembly. Host EVs released by infected host cells contain pathogen as well as host-derived molecules. In this minireview, we summarize the emerging roles of bacterial- and host- derived vesicles in the pathophysiology of invasive pneumococcal infections. Further, we also highlight the translational potential of the vesicles to be developed as vaccines, biomarkers and therapeutics.

## Pneumococcal Membrane Vesicles- Characteristics and Composition

Bacterial outer membrane vesicles are nanometer sized lipid bilayer bound spherical particles formed by pinching of outer membrane in case of Gram-negative bacteria and plasma membrane in case of Gram-positive bacteria. Hence, the Gram-negative vesicles are referred to as outer membrane vesicles (OMVs) and Gram-positive vesicles (MVs) as membrane vesicles. They were first described in *Escherichia coli* as cellular blebs that pinch off from the outer membrane and contain lipopolysaccharide and periplasmic proteins (Work et al., 1966). However, it is now well established that Gram-positive bacteria such as *Bacillus anthracis* (Rivera et al., 2010), *Staphylococcus aureus* (Gurung et al., 2011 *)* and *Streptococcus pneumoniae* (Olaya-Abril et al., 2014) can also shed MVs. *S. pneumoniae* produces numerous MVs ranging between 20-250 nm that contain transmembrane proteins along with cytosolic proteins. Various mechanisms have been proposed for pneumococcal MV release (Brown et al., 2015). The presence of a thick peptidoglycan layer in Gram-positive bacteria acts as a physical barrier for vesicle release. Electron microscopy studies revealed that the vesicles accumulate as internal blebs of membrane invaginations near the division septum where the cell wall is relatively thinner and are thus released during cell division. This is in agreement with the high rates of vesicle release by *S. pneumoniae* during the exponential growth phase as compared to lag or stationary phases (Olaya-Abril et al., 2014). Bacterial MVs have also been shown to fuse together to form nanotubes forming intercellular connections that mediate cargo exchange between neighbouring bacterial cells (Baidya et al., 2018).

Proteomic analysis of the MVs isolated from five different pneumococcal serotypes revealed that they contained several membrane-associated proteins such as PspA, sialic acid ABC transporters, capsular biosynthesis proteins, penicillin-binding proteins, maltose ABC transporters and Foldase protein PrsA, reflective of their membrane origin (Olaya-Abril et al., 2014). The Foldase protein, PrsA is a lipoprotein ubiquitously expressed in Gram-positive bacteria that enables the post-translocational folding of many secreted proteins including enzymes involved in cell-wall biogenesis, virulence factors and toxins. MVs isolated from TIGR4 strain of serotype 4 were enriched for membrane-bound choline-binding proteins, but only had few LPxTG peptidoglycan-anchored proteins and were completely devoid of cell wall lipotechoic acid, suggesting that the MVs bud off from the cell membrane and released through the peptidoglycan layer (Codemo et al., 2018). Lipidomics analysis revealed that despite their origin, pneumococcal MVs are enriched in lipoproteins and short-chain fatty acids as compared to the cell membrane. The higher proportion of unsaturated short chain fatty acids contributes to higher fluidity of the vesicle membrane and facilitates vesicle budding from the plasma membrane (Mercier et al., 2012). Pneumococcal MVs contain several antigenic virulence factors such as the pore-forming toxin pneumolysin (PLY), pyruvate oxidase SpxB, IgA protease, metalloprotease ZmpB, metal ion and sugar transporters and host-adhesion proteins (Olaya-Abril et al., 2014, Choi et al., 2017, Codemo et al., 2018). These proteins are highly immunogenic and accordingly immunization of mice with MVs elicits neutralizing antibodies that protect against subsequent infections (Choi et al., 2017). The toxin, PLY is a cytoplasmic protein lacking a defined export signal and is considered to be released from dead cells upon autolysis. Active toxin export through bacterial vesicles could be an alternative mechanism for PLY release as it has been reported for other bacterial toxins such as staphylococcal alpha toxin and anthrax (Bomberger et al., 2009; Rivera et al., 2010; Thay et al., 2013). Comparative analysis of the composition of MVs from different Gram-positive bacteria revealed that there is little overlap between their protein content (Lee et al., 2009; Rivera et al., 2010; Jiang et al., 2014) and hence their unique protein content could be utilized to develop vesicle-based diagnostic kits for infectious diseases.

## Role of Bacterial Membrane Vesicles in Pneumococcal Infections

**Figure 1:**
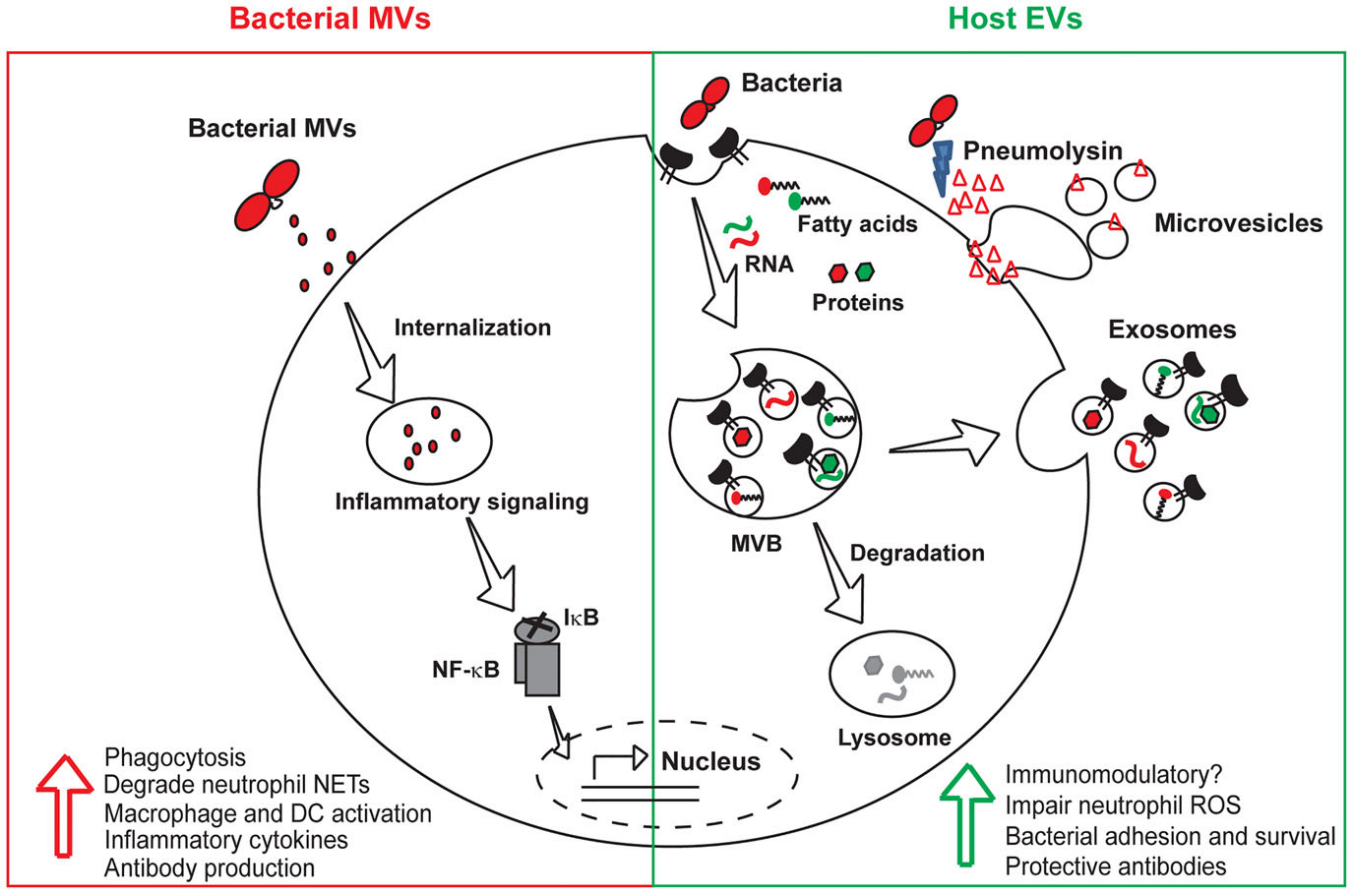
Roles of bacterial and host vesicles in pneumococcal infections. Both bacterial and host-derived vesicles are released during infection. Bacterial MVs containing virulence factors and toxins are internalized by host cells, activate macrophages and dendritic cells resulting in the production of inflammatory cytokines and protective antibodies. Host EVs majorly consist of exosomes derived from exocytosis of multivesicular bodies and microvesicles that directly pinch off from the cell membrane. EVs released from infected host cells harbor bacterial and host macromolecules such as RNA, fatty acids and proteins, that enhance bacterial invasion and intracellular survival by transmitting virulence factors to bystander cells. Pores induced by the pore-forming toxin, pneumolysin are extruded as microvesicles during the process of eukaryotic cell membrane repair. The immunomodulatory role of host EVs is unclear and needs to be further investigated. EVs, host extracellular vesicles; MVs, bacterial membrane vesicles; MVB, multivesicular body; NF-κB, nuclear factor κB; IκB, inhibitor κB; DC, dendritic cell.

Bacterial vesicles (MVs and OMVs) are known to interact with various host cells such as epithelial cells (Codemo et al., 2018, Mehanny et al., 2020; Tiku et al., 2021), neutrophils (Letsiou et al., 2021), macrophages (Jager et al., 2014), dendritic cells (Codemo et al., 2018; Mehanny et al., 2020), B-cells (Vidakovics et al., 2010) and T-cells (Athman et al., 2017). The uptake of bacterial OMVs by host cells has been studied in great detail and has been reported to occur through several mechanisms such as macropinocytosis (Kaparakis-Liaskos and Ferrero, 2015), clathrin mediated endocytosis (Parker et al., 2010), caveolin mediated endocytosis (Sharpe et al., 2011), or lipid-raft mediated endocytosis (Mondal et al., 2016). Pneumococcal MVs have been shown to induce the production of inflammatory cytokines such as IL-6, IL-8, and TNF-α by human dendritic cells (Codemo et al., 2018; Mehanny et al., 2020). However, the impact of pneumococcal MVs on dendritic cell functions such as antigen-presentation, migration and activation of T-cell responses remain to be explored. A recent study showed that pneumococcal MVs activate NF-κB signaling in macrophages (**Figure 1**) and significantly increased the population of M2-polarized splenic macrophages along with increased germinal center formation in mice, indicating activation of both the innate and adaptive immune systems (Yerneni et al., 2021). Streptococcal MV-associated DNase, TatD has been reported to degrade neutrophil extracellular traps and thereby prevent bacterial entrapment by neutrophils (Jhelum et al., 2018). Besides, the pneumococcal MVs have also been shown to bind and scavenge several complement proteins such as C3, C5b-9 and factor H, thereby reducing bacterial complement deposition and opsonophagocytic killing by macrophages (Codemo et al., 2018).

Bacterial MVs have been tested for their immunogenicity to be used as potential vaccine candidates for pneumococcal infections. MVs released by *S. pneumoniae* have been shown to activate the complement system (Codemo et al., 2018). Pneumococcal MVs were found to be immunoreactive to sera from infected patients and to also elicit robust IgG titers in immunized mice resulting in protection against subsequent challenge (Olaya-Abril et al., 2014). Another study found that immunization with bacterial MVs isolated from a non-pathogenic *S. pneumoniae* strain also elicited limited cross-protection against heterologous challenge with a pathogenic strain. However, the presence of the pore-forming toxin, PLY in pneumococcal MVs poses serious risk of hemolysis and this can be addressed by strains expressing genetically modified toxoid derivatives. The PLY toxoid derivative, Pdb (W433F) harboring a point mutation in the membrane binding domain is non-toxic, while retaining immunogenicity and confers protection against many serotypes (Alexander et al., 1994). Hence, further studies are required to throughly investigate the safety of the pneumococcal MVs through large scale clinical trials since the MVs harbor bacterial virulence factors that interfere with the immune system functions (Codemo et al., 2018; Jhelum et al., 2018).

## Role of Host Extracellular Vesicles in Pneumococcal Infections

Mammalian cells release a variety of EVs such as exosomes, microvesicles and apoptotic bodies depending upon their size, composition and biogenesis (**Figure 1**). The exosomes and microvesicles share common biogenesis proteins such as the endosomal sorting ESCRT proteins (Alix, TGS101), tetraspanin proteins (CD9, CD63, CD81) and and their membranes are enriched in cholesterol, sphingolipids, phosphatidylserine and sphingomyelin. Exosomes released by infected cells contain pathogen associated molecular patterns such as bacterial cell wall components (Beatty et al., 2001), proteins, nucleic acids, lipopolysaccharides as well as cytolytic toxins (Husmann et al., 2009). The exosomal route for release of bacterial toxins from infected cells acts as a double-edged sword in a tug of war between the host and the pathogen. While the host cells extrude the portion of the membrane-containing pore through sequential endocytosis and exocytosis during EV release, they also allow for distal spread of toxins inside the host. Host cells are also known to possess membrane repair mechanisms that involve plugging of PLY-induced pores by the annexin proteins and expelling of the pre-formed pores as microvesicles (Wolfmeier et al., 2016). The released microvesicles contain PLY and are therefore likely to induce an immunomodulatory response in the host (**Figure 1**). In agreement, microvesicles shed from PLY-induced human lung epithelial cells were shown to inhibit neutrophil respiratory burst and are enriched in mitochondrial cargo (Letsiou et al., 2021). Proteomic analysis of the PLY-induced microvesicles revealed that they contain a complex protein cargo enriched in annexins and other calcium binding proteins. Besides, they also contain actin-binding proteins, vesicular trafficking ESCRT complex and heat shock proteins. Macrophages infected with *S. pneumonia*e showed a temporal increase in the release of CD63^+^ CD81^+^ microvesicles (Volgers et al., 2017). Pre-treatment of naïve macrophages with EVs isolated from infected macrophages enhanced bacterial adhesion and induced tolerance towards secondary infection. *S. pneumoniae*–induced host EVs did not induce a pro-inflammatory response in contrast to EVs released by cells infected with Gram-negative bacteria such as *P. aeruginosa* and *H. influenzae*. This could be due to the highly immunostimulatory lipopolysaccharide molecule present in the host EVs released during Gram negative bacterial infection, which could be antagonized by polymyxin B treatment. Another study found that dendritic cells, constitutively release an exosome-associated glycoconjugate that is cross-reactive with the capsular polysaccharide antigen of *S. pneumoniae* serotype 14 (Colino and Snapper, 2007). Immunization with the dendritic cell exosomes induced protective antibody response against subsequent infection with serotype 14 strain. The functions of bacterial MVs and host-derived EVs produced during pneumococcal infections are summarized in **Table 1**.

**Table 1:** Characteristics and functions of bacterial and host vesicles in pneumococcal infections.

Composition	Internalization	Immunomodulation	References
**Bacterial MVs**			
Heterogeneous morphology and composition of MVs; contain several immunogenic proteins- MalX, AliA, Ami, PspA, Eno, ABC-SBP, Sphra, and ZamB; enriched in short-chain saturated fatty acids (C12, C14, C15, C16); no lipoteichoic acid; enriched in lipoproteins and transmembrane proteins; vesicle secretome harbors DNase activity	MVs internalized into human somatic cells (lung epithelial cells, keratinocytes) and immune cells (dendritic cells and macrophages)	No cytotoxicity of MVs; MV-immunized mice had robust IgG response and protected against challenges with homologous and heterologous strains; immunoreactive against sera from infected patients; MV-associated DNase, TatD degrades neutrophil NET traps.	(Choi et al., 2017); (Mehanny et al., 2020); (Olaya-Abril et al., 2014); (Jhelum et al., 2018)
Enriched in pneumococcal pore-forming toxin, PLY and choline binding surface proteins like PspC	MVs internalized into human lung epithelial cells and monocyte-derived dendritic cells	Marginal cytotoxicity of MVs that was PLY dependent; MVs activate dendritic cells to upregulate MHC II, CD-86 and release cytokines (IL-6, IL-8, IL-10 & TNF); sequester complement proteins and block bacterial complement deposition; activates NK-κB signalling in macrophages; promotes immune cell infiltration and germinal centre formation in mice; induced arginase-1 and IL-10 expressing alternatively-activated macrophages; vesicle associated PLY promotes bacterial phagocytosis and enhanced survival in macrophages.	(Codemo et al., 2018); (Yerneni et al., 2021)
**Host EVs**			
Microvesicles from PLY stimulated HEK-293 cells were enriched in calcium-dependent proteins (annexin family), cytoskeletal proteins (actin-binding), vesicular trafficking (ESCRT complex), heat shock/chaperone associated, tetraspanins, mitochondrial and cytosolic enzymes; microvesicles from alveolar epithelial cells enriched in mitochondrial cargo.	*S. pneumoniae*-infected alveolar epithelial cells release Annexin/EPCAM/and PLY-containing microvesicles that are internalized by human neutrophils	PLY-induced pores are actively released as microvesicles during plasma membrane repair and impair oxidative burst upon uptake into human neutrophils	(Wolfmeier et al., 2016); (Letsiou et al., 2021)
*S. pneumoniae*-infected macrophages release CD63^+^/CD81^+^ microvesicles		Microvesicles from *S.pneumoniae* -infected macrophages induced modest release of proinflammatory cytokines-TNF-α, IL-8 and IL-1β; increased bacterial adhesion and intracellular survival	(Volgers et al., 2017)
Bone marrow derived dendritic cells constitutively released CD9-containing exosomes		Bone marrow derived dendritic cells constitutively released exosomes containing a glycoconjugate cross-reactive with *S. pneumoniae* capsular polysaccharide type 14; stimulates protective IgG and IgM responses and survival against lethal infection	(Colino and Snapper, 2007)

## Clinical Applications of Vesicles in Pneumococcal Diseases

### Diagnostic Biomarkers

EVs produced during infection have tremendous potential to be developed as biomarkers, drug delivery vehicles and vaccine candidates. There is an urgent need to develop novel rapid diagnostic test for pneumonia and other invasive pneumococcal diseases since the currently available sputum and PCR tests are time consuming and inconclusive (Garnacho-Montero et al., 2018). The inherent nature of the infection-induced EVs to express both host and pathogen borne molecules as well as their presence in body fluids such as blood, bronchoalveolar lavage, saliva and urine makes them ideal candidates for finding novel biomarkers (Schorey et al., 2015). Host EVs also bear unique signature molecules depending upon the tissue of origin that could be used to discriminate between the healthy and diseased tissues. The expression of tissue factor, phosphatidylserine and microRNAs in EVs sampled at the time of intensive care unit admission have been used to discriminate between survivors and non-survivors in viral pneumonia (Rondina et al., 2016) and acute respiratory distress syndrome (Shaver et al., 2017). In this regard, large randomized controlled trials are required to identify and validate suitable biomarkers for pneumoccal infections. The presence of bacterial vesicles in infected patients could reveal the identity of the infecting pathogen as well as determine the presence of virulence determinants such as antibiotic resistance genes. The challenge is to develop highly sensitive techniques to screen bacterial vesicles from patient biofluids due to their low abundance and contamination with host EVs. Bacterial vesicle-based diagnostic sensors could be developed to discriminate between viral, bacterial and non-infectious causes of pneumonia such as lung cancer to decide appropriate treatment strategies.

## Bioengineered Vesicles as Therapeutics

### Host-Derived Vesicles as Targeted Drug Delivery Vehicles

Host EVs can be engineered for drug delivery by exogenously loading them with the desired drugs or biomolecules. Drugs can be either attached to the EV-lipid bilayer by passive incubation (Fuhrmann et al., 2015) in case of hydrophobic compounds or incorporated into the EVs upon permeabilization or electroporation (De Jong et al., 2019) in case of hydrophilic compounds. Porphyrins have been successfully incorporated into EVs using the above techniques and found to have higher activity as compared to the free drug (Fuhrmann et al., 2015). Platelet-derived EVs loaded with anti-inflammatory compounds have been used to treat cytokine storm in pneumonia patients (Ma et al., 2020). The EV content can also be modified by fusion with biosynthetic liposomes carrying both membrane and soluble cargo (Piffoux et al., 2018). The EVs can be functionalized with specific receptors to enable tissue-specific delivery of the loaded cargo. In this aspect, a recent study used integrin β4-expressing exosomes to specifically deliver cancer suppressing microRNA to lung cancer cells *via* interaction with the surfactant protein (Nie et al., 2020). To promote higher bioavailability and bypass off-target effects of engineered therapeutic EVs in the lungs, nebulized EVs can be intranasally administered for direct delivery to the lungs. In a recent study, lung-spheroid cell derived EVs have been successfully administered by inhalation in mouse models to treat lung injury and promote regeneration of alveolar structure (Dinh et al., 2020). The major challenges in EV-based therapeutics are the inherent particle heterogeneity, batch to batch variations, and cost-effective large scale production of EVs.

## Bacterial Vesicles as Vaccine Candidates

The currently available pneumococcal vaccines are the capsular polysaccharide vaccine, PPSV23 and the conjugate vaccine, PCV13 which offer serotype-limited protection against 23 and 13 capsular variants out of the 100 known serotypes respectively and have many shortcomings such as increased prevalence of non-vaccine serotypes due to capsule switching (Chang et al., 2015) and high recombination frequency in pneumococci (Mostowy et al., 2017). Therefore, there is an urgent need to develop new vaccines that offer wide protection against several serotypic variants (Berical et al., 2016). Bacterial vesicles are promising candidates for novel vaccine candidates against pneumococcal and other respiratory pathogens since they harbor several antigenic molecules from the pathogen and are lesser virulent compared to the live organisms (Behrens et al., 2021). The major challenge with vesicle-based vaccines is to elicit a sufficient immune response without eliciting undesired cytotoxic effects. In this regard, vesicles from non-pathogenic bacterial species can be engineered to express heterologous antigens from virulent pathogens or attenuated endotoxins molecules. OMVs from engineered *E. coli* strain expressing Streptococcal antigens fused to OmpA protein were immunogenic and shown to protect against Streptococcal infections (Fantappie et al., 2014). Glycoengineered OMVs from non- pathogenic *E. coli* strain, CLM37 expressing capsule glycan from *S. pneumoniae* serotype 14 were effective in generating protective antibodies against pneumococci (Valguarnera and Feldman, 2017). OMVs from the Gram-negative bacterial pathogen, *Neisseria meningitis* have been genetically engineered to express attenuated endotoxin and successfully used against meningococcal infections (Beernink et al., 2019). Another limitation concerning the use of bacterial vesicles as potential vaccines is the low yield and problem with large scale production. Hypervesiculating bacteria have been genetically engineered by altering peptidoglycan cross-linking or mutations in proteins linking the membrane to peptidoglycan (Van De Waterbeemd et al., 2010). The greatest challenge with regards to clinical translation of bacterial vesicles is the standardization of scalable vesicle isolation procedures. It is therefore important to develop standardized techniques for consistency in vesicle isolation and purification and develop technologies for large scale production of EVs for clinical application. In this regard, the Generalized Module for Membrane Antigens technology is an attractive vaccine strategy that enables production of multivalent safe, affordable and effective vaccines (Micoli et al., 2020). In this technique, OMVs are derived from strains engineered to over-vesiculating phenotype and harboring mutations in lipopolysaccharide genes. This allows the faithful presentation of multiple antigens in their native state resulting in good immunogenicity. They can be purified to high yield and purity and have been used to develop affordable vaccines for many bacterial pathogens like Shigella (Launay et al., 2017), Neisseria (Koeberling et al., 2014) and Salmonella (Rossi et al., 2016).

## Conclusions

Bacterial and host vesicles play important roles during invasive pneumococcal diseases and influence the host response to infection (**Figure 1**). Bacterial vesicles express pathogenic antigens and hold promise as novel vaccine candidates. Vesicle-based vaccines are safer in comparison to whole organism vaccines and don’t require extensive cold-chain logistics due to their thermostability. However, standardized GMP procedures for large scale production of bacterial MVs need to be developed. Host-derived EVs released from infected cells contain both pathogenic and host molecules and can be exploited to develop biomarkers to discriminate the pathogenic organism and predict the disease severity in patients. The host-derived EVs can also be engineered to precisely deliver therapeutic payloads to target cells and modulate the host immune response. However, the safety and off-target effects of the engineered EVs needs to be extensively tested using large scale clinical trials before it can be developed to clinical therapies.

## Author Contributions

KS formulated the concept for the review. KS and SP performed the literature review, prepared figure and table. KS critically revised the article before final submission. All the authors have read the final submitted version and approved it for publication.

## Funding

KS received extramural funding through the DST-INSPIRE Faculty fellowship (DST/INSPIRE/04/2019/002238) and DST-SERB Start-up Grant (SRG/2021/000401) Department of Science and Technology, India, DBT-Ramalingaswami Re-entry fellowship (No. BT/RLF/Re-entry/46/2020) from the Department of Biotechnology, India and intramural funding from Rajiv Gandhi Centre for Biotechnology, Trivandrum.

## Conflict of Interest

The authors declare that the research was conducted in the absence of any commercial or financial relationships that could be construed as a potential conflict of interest.

## Publisher’s Note

All claims expressed in this article are solely those of the authors and do not necessarily represent those of their affiliated organizations, or those of the publisher, the editors and the reviewers. Any product that may be evaluated in this article, or claim that may be made by its manufacturer, is not guaranteed or endorsed by the publisher.
